# Atypical Femur Fractures in Patients Treated with Bisphosphonates: Identification, Management, and Prevention

**DOI:** 10.5041/RMMJ.10259

**Published:** 2016-10-31

**Authors:** Judith Sarah Bubbear

**Affiliations:** Department of Rheumatology, Whipps Cross University Hospital, Barts Health NHS Trust, London, UK

**Keywords:** Atypical femoral fracture, bisphosphonate, drug holiday, osteoporosis

## Abstract

Osteoporosis is a common condition with significant health care costs. First-line therapy is with bisphosphonates, which have proven anti-fracture efficacy. Around 10 years after the introduction of bisphosphonates reports began to be published of atypical femoral fractures (AFFs) that may be associated with this therapy. These fractures are associated with significant morbidity although lower mortality than the more common osteoporotic neck-of-femur fractures. A case definition has been described to allow identification of this class of fracture. Further work has established a high relative risk of AFFs in patients treated with bisphosphonates, but a low absolute risk in comparison to that of osteoporotic fractures. Proposed pathological mechanisms include low bone turnover states leading to stress/insufficiency fractures. Clinicians should be aware of the risk of AFFs and in particular the high rate of prodromal thigh/groin pain that warrants investigation in a patient receiving a bisphosphonate. If an incomplete fracture is diagnosed then bisphosphonate therapy needs to be stopped and prophylactic surgery may be considered. Due to these rare side effects patients on bisphosphonates require regular review, and this is particularly advised after 5 years of oral or 3 years of intravenous therapy.

## BACKGROUND

Osteoporosis is a skeletal disorder characterized by compromised bone strength predisposing to an increased risk of fracture.^1^ It is common with an increasing prevalence with age and a greater incidence in post-menopausal women than in men. In 2010 the prevalence in the 27 countries of the EU was approximately 27.6 million.^2^ In the United States 10 million people over the age of 50 have osteoporosis.^3^ The consequence of osteoporosis is fragility fractures that cause significant morbidity as well as mortality. Worldwide there is a fracture almost every 3 seconds, resulting in 8.9 million fractures annually; 1 in 3 women and 1 in 5 older men will suffer a fragility fracture after the age of 50 with a financial burden that is comparable to other chronic conditions such as asthma, hypertensive heart disease, or rheumatoid arthritis.

The bisphosphonates (BP) are analogues of inorganic pyrophosphate. Bisphosphonates act by inhibiting osteoclast activity and thereby inhibit bone resorption. These drugs are commonly prescribed for osteoporosis and have been shown in good-quality randomized controlled trials to reduce the risk of fragility fractures at vertebral, non-vertebral, and hip sites (Table 1). These studies are mostly of 3–4 years’ duration and show fracture reduction rates of 40%–70% in vertebral fractures, 15%–39% in non-vertebral fractures, and 20%–50% in hip fractures.^4^–^7^ On the basis of this evidence, BPs are considered first-line therapy for patients requiring treatment for osteoporosis and are also used in other conditions such as Paget’s disease of bone, malignancies involving bone, and tumor-induced hypercalcemia. In addition to the anti-fracture efficacy a reduction in mortality has also been reported in patients treated with intravenous BP following a hip fracture.^8^

**Table 1:** Anti-Fracture Efficacy of Anti-Resorptive Treatments for Postmenopausal Women with Osteoporosis when Given with Calcium and Vitamin D.

Drug	Vertebral Fracture	Non-Vertebral Fracture	Hip Fracture
Alendronate	A	A	A
Ibandronate	A	A*	NAE
Risedronate	A	A	A
Zoledronic acid	A	A	A
Denosumab	A	A	A

* Subsets of patients only (*post hoc* analysis).

A, anti-fracture efficacy; NAE, not adequately evaluated.

The first report of a possible link between BP use and “atypical fractures” of the femoral shaft was published by Odvina et al. in 2005.^9^ They reported nine patients who had sustained non-traumatic non-vertebral fractures whilst taking alendronate. Some patients were also taking estrogen and/or glucocorticoids. After this report followed many other case reports of similar fractures. There was often little or no trauma involved in these fractures, and over 70% were preceded by thigh or groin pain.^10^

These cases caused great anxiety for patients and clinicians alike, and therefore the American Society of Bone and Mineral Research (ASBMR) convened a taskforce in 2009 to investigate this area and examine the evidence in a systematic manner.^10^ They repeated this in 2013, publishing an updated report.^11^

## CASE DEFINITION

The ASBMR task force reviewed and summarized the available evidence, and this has led to the agreement of a case definition of an atypical femoral fracture (AFF). The aim of this was to identify AFFs as distinct from typical neck-of-femur fractures that are seen with osteoporosis, and femoral shaft fractures that are seen with high-trauma injuries. With a case definition having been established, this has guided ongoing research and aided identification of this cohort of patients.

It is worthy of note that there are cases of AFF in all case series in individuals who have not been exposed to bisphosphonates.^12^

The classification of a femoral fracture as an AFF specifically excludes high-trauma fractures, fractures of the neck of femur, intertrochanteric fractures with subtrochanteric spiral extension, and pathological fractures associated with primary or secondary metastatic bone disease or other metabolic bone diseases. The case definition was honed in the 2013 ASBMR report to clarify features that distinguish AFFs from ordinary osteoporotic fractures of the femur. A diagnosis relies on having a least four of five major features, with the major features being: minimal trauma, fracture originates laterally and is transverse, complete fractures may have medial spike, incomplete only involve lateral cortex, the fracture is non-comminuted or minimally comminuted, and the presence of localized periosteal or endosteal thickening of the lateral cortex (“beaking” or “flaring”). There are associated minor features, none of which was required to make a diagnosis, but which have been associated with AFFs. Localized periosteal (“beaking” or “flaring”) or endosteal thickening of the lateral cortex at the fracture site was added to the case definition 2013 as that had recently been reported. See Table 2 for case definition.

**Table 2:** The American Society for Bone and Mineral Research Task Force 2013 Revised Case Definition of Atypical Femoral Fractures.

ASBMR: Definition of AFF
To satisfy the case definition of AFF, the fracture must be located along the femoral diaphysis from just distal to the lesser trochanter to just proximal to the supracondylar flare In addition, at least four of five major features must be present; none of the minor features is required, but they have sometimes been associated with these fractures
Major Features Minimal trauma Fracture originates laterally and is transverse Complete fractures may have medial spike; incomplete only involve lateral cortex Non-comminuted or minimally comminuted Localized periosteal or endosteal thickening of the lateral cortex (“beaking” or “flaring”)
Minor Features Generalized increase in cortical thickness of femoral diaphyses Unilateral or bilateral prodromal symptoms—dull ache in groin or thigh Bilateral incomplete or complete femoral diaphysis fractures Delayed fracture healing
Excludes Fractures of the femoral neck Intertrochanteric fractures with spiral subtrochanteric extension Periprosthetic fractures Pathological fractures associated with primary or metastatic bone tumors and miscellaneous bone diseases (e.g. Paget’s disease, fibrous dysplasia)

Taken from: Shane E, Burr D, Abrahamsen B, et al.^11^ Reproduced with permission of John Wiley & Sons, © 2014 American Society for Bone and Mineral Research.

## EPIDEMIOLOGY

The best-quality epidemiological evidence comes from studies with radiographic adjudication of the fractures as definite AFFs. Relative risk of BP use in AFF is very variable in different studies, ranging between 2- and 128-fold.^13^,^14^ However, the absolute risk is much lower. Again, this varies between studies, but ranges between 3.2 and 50 cases per 100,000 person years.^15^,^16^ A recent systematic review has reported an adjusted odds ratio of 2.71 for subtrochanteric fractures in patients taking bisphosphonates.^17^

Some but not all studies suggest an increase in incidence with duration of therapy—e.g. one study reported an increase in age-adjusted incidence from 1.8/100,000/year with 2 years of treatment to 113/100,000/year with 8–9.9 years of treatment.^18^ Meier et al. reported an exposure of 5–9 years being associated with a greater risk of AFF OR of 117.1.^16^ Schilcher et al. reported in 2014 that use of a bisphosphonate for over 4 years increased the relative risk to 126, with an absolute risk of 11/10,000 person years.^19^

The 2010 ASBMR task force report examined all the evidence and concluded that the incidence of AFF was very low, particularly in light of the large numbers of fractures that are prevented by bisphosphonate use, and also that a causal link to bisphosphonates had yet to be proven. Again in 2013 they concluded that there was still no evidence for a causal relationship, but that the “fairly consistent magnitude of association” is unlikely to be accounted for by confounders that may be currently unknown or unmeasured. Further weight is given to potential causality by evidence that stopping treatment with a bisphosphonate reduces the risk going forward of AFFs with a 70% reduction in AFFs one year after stopping therapy.^19^

It is worthy of note that AFFs have also been described in patients treated with denosumab (monoclonal antibody against receptor activator of nuclear factor kappa B ligand), which is also a potent inhibitor of bone resorption.^20^,^21^ Incidence of AFF appears to be similar to that seen with bisphosphonate therapy, although no study has made a direct comparison between bisphosphonates and denosumab. Approximately 70% have a history of prodromal thigh or groin pain, 28% had bilateral fractures and bilateral radiographic abnormalities, and 26% had delayed healing; 34% had concomitant glucocorticoid use.^10^

It has been suggested that the risk of AFFs may be high in individuals taking glucocorticoids,^22^ proton pump inhibitors,^23^ and in patients with rheumatoid arthritis or diabetes. Other reported potential risk factors include genu varus, varus/bowed femur,^24^ and collagen disease.^25^

The risks appear to be increased in patients of Asian origin. A recent paper showed an 8-fold increased risk in individuals from an Asian background.^26^ In addition one study showed a particular increase in AFFs in individuals treated with a bisphosphonate with low bone mass (osteopenia) in comparison with those with osteoporosis.^27^

A recent paper has reported two cases of AFF seen in patients where radiographs taken prior to bisphosphonate therapy showed localized cortical thickening, which raises the possibility that AFFs occur at the sites of pre-existing stress or insufficiency fractures.^28^

Although AFFs are associated with significant morbidity the mortality rates are significantly lower than seen in osteoporotic neck of femur fractures, with a one-year reported mortality of 2.4% in comparison to 9.6% among women aged 70–75 in a comparable Swedish cohort.^29^,^30^

## PATHOGENESIS

A number of possible mechanisms have been put forward to explain the pathogenesis of these fractures. Currently this area remains largely unclear. There are commonalities between AFFs and stress fractures. A stress fracture is caused by abnormal loading of normal bone, whereas an insufficiency fracture is caused by normal loading of an abnormal bone. Both these types of fractures are most common in the lower limbs due to the increased loading. In a stress fracture microcracks appear that over time coalesce and without repair progress to a fracture. The current consensus of the ASBMR task force is that AFFs are stress or insufficiency fractures that progress over time.^11^

There have been long-standing concerns that by reducing bone turnover the bisphosphonates may lead to brittle bones. The features of a transverse fracture and the lack of comminution are features of fractures in brittle bones. Almost all biopsy studies in AFFs reveal reduced or absent osteoblasts and osteoclasts and little or no tetracycline double-labeling, which would indicate suppression of bone remodeling.^10^ This may be expected in patients taking a bisphosphonate, and not all biopsies were from the fracture site, with some being iliac crest biopsies. However, the suggestion is that bone turnover is suppressed, potentially causing insufficiency fractures under normal loading conditions.

Further theories have been put forward that include the effects of bisphosphonates on the collagen in the organic matrix of bone,^31^ effects on angiogenesis,^32^ the healing of fatigue fractures, and a reported influence of the lower limb geometry on the stress generated on the lateral aspect of the femoral cortex.^33^,^34^

## MANAGEMENT OF AFFS

It appears that these AFFs are stress fractures that develop over time.^35^ Initially one can identify a cortical bump that is thought to represent early periosteal thickening, then a transverse cortical lucency develops, which may progress to become a complete fracture.

**Figure 1:**
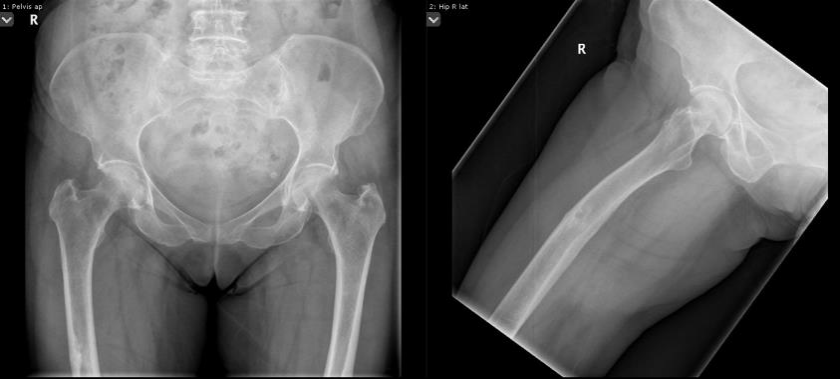
Radiograph Showing Incomplete Atypical Femoral Fracture of the Right Femur with Lateral Cortical Thickening.

**Figure 2:**
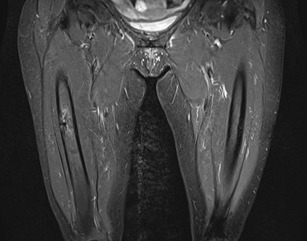
MRI Scan Showing Incomplete Atypical Femoral Fracture with Edema and a Lateral Fracture Line.

**Figure 3:**
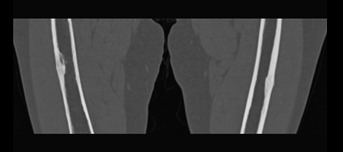
CT Scan Showing Bilateral Lateral Cortical Thickening.

More than half of the patients experience prodromal thigh/groin pain.^9^ Health professionals need to be aware of AFF as a potential complication of BP use and the potential significance of thigh pain. Patients on BP (or other anti-resorptive drugs) should be routinely asked about thigh/groin pain, and patients should also be educated to be aware of these symptoms. If symptoms are reported then a plain radiograph of the femur should be obtained (Figure 1). If the radiograph has features to suggest incomplete fragility fractures, such as cortical thickening, then further imaging should be obtained. Ideally magnetic resonance imaging (MRI) should be performed, which allows assessment of a cortical fracture line, associated bone marrow edema, or hyperemia suggestive of a stress fracture (Figure 2). A cortical lucency would suggest an incomplete AFF, whereas purely edema would be more in keeping with a stress reaction. If MRI is not possible computerized tomography (CT) can also help and would detect fracture lines and new bone formation (Figure 3). In addition, incomplete fractures will be metabolically active on isotope bone scanning, although this will not demonstrate a fracture line.

It is essential that imaging of the contralateral femur, even if asymptomatic, is also carried out, as these fractures are often bilateral.

If an incomplete fracture is detected the BP should be stopped immediately. Patients should receive adequate calcium and vitamin D. If there is pain, then orthopedic intervention with intramedullary nailing is advisable to prevent progression to a complete fracture. If there is no pain, then management can be conservative with reduced weight-bearing for 2–3 months. If there is no improvement, then prophylactic nailing should be considered.^11^

Teriparatide (1–34 parathyroid hormone analogue, TPD) has been used as a treatment to improve fracture healing in cases of AFF. This is the only currently available anabolic agent for bone. Systematic review has shown positive outcomes for fracture healing with the use of TPD after AFF.^36^ Histology shows increased bone formation in patients treated with TPD for AFFs.^37^ Although no randomized controlled trial data are currently available for TPD it seems reasonable to try in patients who fail to heal with conservative therapy. There is an ongoing randomized controlled trial to evaluate this treatment formally.

There are two reported cases of strontium ranelate being used and improving fracture healing.^38^,^39^ Recent warnings about cardiovascular risk may preclude this, but short-term use may be helpful in some individuals, particularly in health economies where it may be difficult to get access to TPD. There is also one reported case in the literature of TPD being used on a weekly rather than daily basis and improving fracture healing, which may be more cost-effective.^40^ In addition one case has been reported of the use of low-intensity pulsed ultrasound, which may prove helpful in patients in whom TPD is contraindicated (e.g. those with malignancy).^41^

After an AFF the options for treatment of osteoporosis are TPD or raloxefine (selective estrogen receptor modulator); these have not been associated with AFF and are not anti-resorptive in mode of action.

## PREVENTION OF AFFS

These rare complications and others such as osteonecrosis of the jaw have led to a change in practice with regard to long-term BP therapy. It is now advised that BP therapy should be regularly reviewed with the aim of stopping treatment in patients who no longer require it and thereby reducing the risk of complications of long-term treatment. The optimal duration of treatment with a BP is not known.

Bisphosphonates strongly adhere to hydroxyapatite on the bone surface and therefore have a persistent effect even when therapy is stopped. Two published studies have evaluated the long-term use of BPs. The FLEX study was a 5-year extension of the pivotal fracture prevention study with alendronate and showed a perseverance of fracture risk reduction for 2 years after a 5-year course of treatment.^4^ An extension of the HORIZON study compared the use of 3 years of intravenous zoledronic acid and stopping treatment, with 6 years of continuous therapy.^42^ It showed that 3 years of treatment gives fracture risk reduction for a further 3 years. This evidence for an ongoing effect of treatment, even when it is stopped, and the risks of long-term side effects have led to the concept of a “drug holiday.”

The UK National Osteoporosis Guideline Group (NOGG) advises a treatment review after 5 years of treatment with oral BPs and 3 years of treatment with zoledronic acid.^43^ They recommend continued treatment in those at high risk, for which they give the examples of: aged 75 or more, previous hip/vertebral fracture, glucocorticoids at a dose of ≥7.5mg daily. The ASBMR has also published a taskforce report on managing osteoporosis patients after long-term BP therapy and recommend that high-risk older women with a low hip T-score or high fracture risk should continue on treatment.^44^ The UK NOGG also recommend a review of treatment in those who sustain one or more fractures when receiving therapy (after checking >80% compliance) and also recommend treatment is continued in patients where the total hip or femoral neck T-score is below −2.5.

The ASBMR suggest that for those women not at high fracture risk after 3–5 years of treatment a drug holiday of 2–3 years could be considered with periodic reassessment, and NOGG state that if treatment is stopped fracture risk should be reassessed if a patient has a new fracture or after 2 years if no fracture occurs. In the case of zoledronic acid 3 years of treatment should be sufficient for the majority of patients.

The United States Food and Drug Administration have added to the “warning and precautions” regarding bisphosphonates and AFFs, advising that patients on bisphosphonates should be reassessed after 3–5 years of therapy.

The ASBMR long-term BP taskforce report makes the valid point that it is unlikely that there will be further future studies to address the question of long-term BP use and therefore we are unlikely to have further data available to make definitive recommendations.^44^

Evidence suggests that stopping a BP will have an influence on the risk of AFF. Schilcher et al. reported on a Swedish cohort of 45 women with confirmed AFFs who then stopped bisphosphonates.^19^ They reported that the risk fell by 70% each year after stopping therapy with the most dramatic risk reduction in the first year. However, it is worth noting that these analyses were based on 46 AFF events with only four occurring after the first year. There was only short-term follow-up, which may lead to the derived estimates being overestimated.

Data from the Kaiser Database suggests that if BP is stopped soon after an AFF then only 20% will fracture the contralateral femur as opposed to 50% if the BP is continued for 3 years after AFF.^45^

## CONCLUSIONS

**Figure 4:**
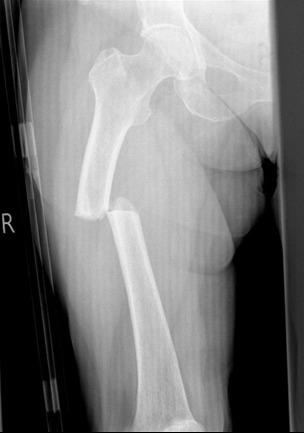
Atypical Femoral Fracture of the Right Femur.

Atypical femoral fractures (Figure 4) are rare, but the evidence when taken as a whole does suggest that their incidence is significantly increased in patients treated with BP therapy. The relative risk may be high, but the absolute risk is low. For the vast majority of patients the reduction in risk of osteoporotic fracture with treatment vastly out-weighs the risks of rare side effects such as AFFs. However, BP therapy should be regularly reviewed, particularly after 5 years, and only continued in those patients deemed to be at high risk. Patients who complain of thigh or groin pain must be investigated for potential incomplete fractures and managed appropriately, in particular having their anti-resorptive therapy stopped. Health professionals need to be aware of risks, but it is important that the comparative risks of treatment versus lack of treatment are conveyed to patients.

## Abbreviations

AFF: atypical femoral fracture
ASBMR: American Society of Bone and Mineral Research
BP: bisphosphonate
CT: computerized tomography
MRI: magnetic resonance imaging
NOGG: National Osteoporosis Guideline Group (UK)
TPD: teriparatide
